# Long‐Term Humoral Immune Response After West Nile Virus Convalescence in Horses in a Geographic Area of Multiple Orthoflavivirus Co‐Circulation

**DOI:** 10.1111/jvim.70176

**Published:** 2025-06-17

**Authors:** Csenge Hanna Tolnai, Petra Forgách, András Marosi, Orsolya Fehér, Bettina Paszerbovics, Miklós Tenk, Zsombor Wagenhoffer, Orsolya Kutasi

**Affiliations:** ^1^ Department of Microbiology and Infectious Diseases University of Veterinary Medicine Budapest Budapest Hungary; ^2^ Health Safety National Laboratory Budapest Hungary; ^3^ Nutrition and Laboratory Animal Science University of Veterinary Medicine Budapest, Institute for Animal Breeding Budapest Hungary; ^4^ Department of Biostatistics University of Veterinary Medicine Budapest Budapest Hungary

**Keywords:** antibody, cross‐reaction, equine, serology

## Abstract

**Background:**

In the last three decades, West Nile virus (WNV, *Flaviviridae, Orthoflavivirus* genus) has become one of the most important encephalitic agents worldwide, causing substantial numbers of cases in humans and horses every year by re‐emerging in endemic areas and emerging in new territories. It is considered that after natural WNV infection, humans and birds develop long‐term immunoprotection, but data on immunoprotection in horses is scarce.

**Hypothesis:**

West Nile virus infection provides long‐term humoral immunity in subclinically infected horses.

**Animals:**

Client‐owned, naturally WNV subclinically infected non‐WNV‐vaccinated, healthy horses.

**Methods:**

In this prospective cohort study, anti‐WNV neutralizing antibody (nAb) titers of 25 horses were monitored for 5 consecutive years in Hungary. Serum samples were collected annually. First, a WNV immunoglobulin G (IgG) ELISA was performed, followed by virus neutralization tests (VNT) for endemic orthoflaviviruses. A VNT titer > 8 was considered positive.

**Results:**

The mean WNV titer of horses was 260.64 ± 336.74 in 2019, 114.32 ± 107.36 in 2020, 95.38 ± 115.56 in 2021, 22.53 ± 25.71 in 2022 and 6.31 ± 5.15 in 2023. A significant decrease (*p* < 0.001) in the nAb titers occurred over time. In 2023, 88% of the horses had WNV VNT titers below the cut‐off value.

**Conclusion and Clinical Relevance:**

Our results showed a significant decrease in WNV titers over time. Because nAbs correlate best with orthoflavivirus protection, our findings suggest that horses might not be protected against re‐infection. We recommend regular nAb titer testing or vaccination in endemic areas.

**Abbreviations**
ADE: antibody‐dependent enhancement
CPE: cytopathic effect
DENV: dengue virus
DMEM: Dulbecco's modified eagle medium
E: envelope protein
ECDC: European Centre for Disease Control
EIA: equine infectious anemia
IP: inhibitory percentage
JEV: Japanese encephalitis virus
mAb: monoclonal antibody
MBC: memory B‐cell
MW‐NT: micro‐well neutralization test
OD: optical density
PFU: plaque forming unit
PI: post‐infection
PRNT: plaque reduction neutralization test
TBE: tick‐borne encephalitis
TBEV: tick‐borne encephalitis virus
TCID50: tissue culture infective doses of 50%
USUV: Usutu virus
VNT: virus neutralization test
WNND: West Nile neuroinvasive disorder
WNV: West Nile virus
WOAH: World Animal Health Organization

## 1 Introduction

West Nile virus is a mosquito‐transmitted member of the *Orthoflavivirus* genus [1]. It was first isolated in 1937 from a febrile woman in Uganda [2]. At the beginning of the 21st century, the virus emerged in the European and American continents and caused major outbreaks of thousands of cases [3, 4]. The *Orthoflavivirus* genus belongs to the *Flaviviridae* family and includes several other relevant pathogens of humans and horses [5]. Presently, three orthoflaviviruses are endemic in Hungary: West Nile virus (WNV), Usutu virus (USUV), and tick‐borne encephalitis virus (TBEV) [6]. Because of climate change and various socioeconomic factors, the co‐circulation of orthoflaviviruses has become an increasing global problem in recent years [7, 8, 9].

West Nile virus is maintained in an enzootic cycle between birds and ornithophilic mosquitoes. Horses and humans serve as incidental hosts. After inoculation, horses develop viremia 1–6 days post‐infection (PI), lasting for 1–3 days with viral titers ranging between 10 and 10^3^ plaque‐forming units/mL [10]. It is estimated that 10%–20% of horses develop neurological signs, with ataxia of different grades, weakness, muscle fasciculations, tremors, and cranial nerve deficits being reported most frequently [10, 11, 12, 13, 14]. Because of the long incubation period (7–10 days) but short‐term, low‐titer viremia in horses, the diagnosis of WNV infections relies on serological methods. Virus‐specific immunoglobulin M (IgM) becomes detectable 5–7 days PI and often is used to verify recent WNV infection. Virus‐specific immunoglobulin G (IgG) can be detected from 10 days PI to at least 1 year PI [15]. The virus‐specific neutralizing antibody response is reported to target the viral surface envelope (E) glycoprotein. However, this protein shares different levels of amino acid sequence homology among individual members of the genus, and consequently, an orthoflavivirus infection elicits not only virus type‐specific but also genus cross‐reactive antibodies [16, 17]. The latter can hamper the diagnosis of the viral agents and influence the clinical manifestation of secondary infections [15]. The overlapping geographic presence of orthoflaviviruses and their cross‐reactive antibodies necessitate understanding of the long‐term immune response and its implications in protection against homologous or heterologous members of the genus.

Currently, the concentration of neutralizing antibodies is the best indicator of protection against orthoflaviviruses [18, 19]. After orthoflaviviral infections, birds and humans develop long‐term immune protection against the pathogens, even in the case of subclinical seroconversion [20, 21]. However, some conflicting results regarding long‐term antibody concentrations after WNV infection in humans have been observed [22, 23, 24]. We investigated the humoral immune response of WNV in naturally subclinically infected horses over a five‐year period in Hungary, where other endemic orthoflaviviruses are present. The primary objective of our prospective cohort study was to assess the long‐term humoral immune response of horses naturally and subclinically infected with WNV and other endemic orthoflaviviruses.

## 2 Materials and Methods

### 2.1 Study Design

After the major WNV outbreak in 2018 in Hungary, a WNV serosurvey was performed in horses in 2019. Blood samples for the study were obtained as part of annual obligatory active equine infectious anemia (EIA) surveillance of registered horses in Hungary. First, a commercially available WNV ELISA was performed. Because only healthy animals with no prior history of neurological signs were enrolled in the study, in the case of a positive WNV IgG result, the owner and the treating veterinarian of the horse were contacted. After confirming the animal's health status, owner consent was obtained, and serum samples were further evaluated using micro‐well virus neutralization tests (MW‐NT) for WNV, USUV, and TBEV. A detailed description of these procedures is given below. Blood samples from the selected horses were taken each year before the mosquito season (from June to October) from 2019 to 2023. Samples of the same year were tested simultaneously for all three orthoflaviviruses each year.

### 2.2 Animals

**FIGURE 1:**
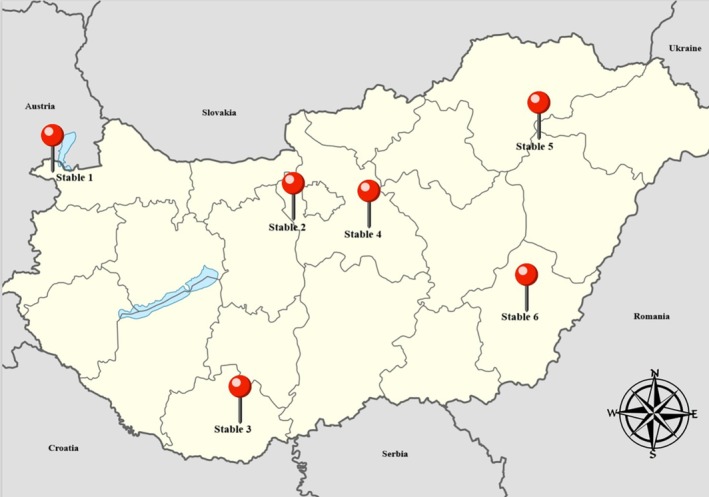
Location of the stables in Hungary. Stable 1—Győr‐Moson‐Sopron County, Stable 2—Pest County, Stable 3—Baranya County, Stable 4—Pest County, Stable 5—Hajdu‐Bihar County, Stable 6—Békés County.

Twenty‐five client‐owned horses with positive initial WNV IgG ELISA results were chosen. The inclusion criteria for animals were: adult, healthy horses with no prior history of neurological signs, and non‐vaccinated for WNV. The animals were kept in 6 different stables: 2 horses in Stable 1 in Győr‐Moson‐Sopron County, 8 horses in Stable 2 in Pest County, 5 horses in Stable 3 in Baranya County, 2 horses in Stable 4 in Pest County, 6 horses in Stable 5 in Hajdú‐Bihar County, and 2 horses in Stable 6 in Békés County. The location of each stable is represented in Figure 1. All animals remained within the same facility during the study. These horses were followed for 5 consecutive years, and their health status and absence of neurological signs were monitored annually.

### 2.3 Sera Samples

Blood samples were taken by jugular venipuncture into tubes without anticoagulants. Samples were transported to the laboratory at 4°C, where serum was separated. Blood samples were centrifuged at 1860 rpm for 10 min, and then sera were transferred to microcentrifuge tubes. Serum samples were stored at −20°C.

### 2.4 Cell Lines and Viruses

African green monkey kidney Vero cells (ATCC, United States) were used for neutralization assays. Cells were grown in Dulbecco's modified eagle medium (DMEM; Capricorn Scientific GmbH, Germany) supplemented with 10% fetal bovine serum (FBS; Capricorn Scientific GmbH, Germany) and 1% penicillin/streptomycin (Sigma‐Aldrich Co., United States) to make complete media. Cells were maintained in a humidified atmosphere at 37°C in 5% CO_2_ and were used to grow virus stocks and determine virus titrations.

West Nile virus strain 578/10 lineage II (GenBank accession number KC496015.1) was used [25]. Usutu virus Budapest‐2005 strain (GenBank accession number EF206350.1) was used [26]. Tick‐borne encephalitis virus KEM‐1 strain (GenBank accession number MW256716.1) was used [27].

All viral suspensions were previously titrated to 100 TCID_50_/mL (tissue culture infective doses of 50%).

### 2.5 Diagnostic Assays

#### 2.5.1 WNV IgG ELISA

Horse serum samples were screened by blocking enzyme immunoassay (INgezim West Nile Compac, Ingenaza, Spain) for IgG antibodies against WNV. This test detects antibodies produced against the E protein of WNV. The ELISA was performed following the manufacturer's protocol. Results were interpreted by calculating the inhibition percentage (IP) as described in the manufacturer's guidelines. An IP ≥ 40% was considered positive, between 30% and 40% was interpreted as inconclusive, and < 30% was considered negative.

#### 2.5.2 Micro‐Well Neutralization Assay

The micro‐well virus neutralization assay was performed on all serum samples according to the World Organization for Animal Health (WOAH) Manual of Diagnostic Tests and Vaccines for Terrestrial Animals (Chapter 3.1.26.), except for the level of the serial dilution. In our study, a two‐fold serial dilution was made from each serum sample, instead of the recommended dilution (1:5 to 1:640) [28]. Briefly, 96‐well cell culture plates (TPP Techno Plastic Products AG, Switzerland) were used, and each well was filled with 50 μL of 0% DMEM (not supplemented with FBS). After inactivation at 56°C for 30 min, a twofold serial dilution of serum samples from 1:2 to 1:2048 was performed. Then, 50 μL of 100 tissue culture infective dose‐50% (TCID_50_/mL) viral suspension was added to the diluted serum samples. After incubation, 10^4^ Vero cells suspended in DMEM with 10% fetal calf serum were added to each well. Plates were incubated for 1 h at 37°C with 5% CO_2_. Back titration of each viral suspension was performed to confirm the viral titers, and non‐infected cell culture was used as a negative control. The plates were incubated and monitored daily for 5 days at 37°C with 5% CO_2_. The presence and extent of cytopathic effects (CPE) were evaluated on the 5th day. The serum neutralizing titer was estimated as the highest serum dilution showing 50% of CPEs. The cut‐off value of the WOAH manual (1:10) was corrected to our dilution. Therefore, sera with neutralizing titers > 1:8 were identified as positive. In case of simultaneous positive results for two or all viruses, the sample was considered positive for the agent with the highest neutralization titer.

### 2.6 Statistical Analysis

In our statistical modeling, titer values were log‐transformed using base 2. We applied a mixed effects model with the log‐transformed titer as the dependent variable. The model included the year as a continuous variable in the fixed effects component. The random effects structure consisted of both a random slope and a random intercept. The random slope allowed for variability in the pattern of antibody changes across individual horses, acknowledging that the rate of change differed. The random intercept accounted for dependency within the data, recognizing that measurements from the same horse were not independent. The statistical analysis was conducted in R (version 4.2.1, 2022‐06‐23 ucrt) using the lme function from the nlme package.

## 3 Results

### 3.1 Animals

Altogether, 125 samples were obtained over the 5‐year study period. During the study, none of the horses were reported to have any clinical signs related to orthoflavivirus infections. The mean age of horses was 11.48 ± 3.92 years (range, 3–22 years) in 2019. The study involved 13 (52%) mares and 12 (48%) geldings. There were 8 Hungarian warmbloods, 3 Hungarian sport ponies, 3 Oldenburgers, 3 Holsteiners, 2 Welsh Ponies, 2 Gidrans, 1 KWPN, 1 Trakhener, 1 Connemara, and 1 German Sport horse included. No correlations were identified between the age or sex of horses and the VNT titers.

### 3.2 ELISA

All horses were WNV IgG positive in the first year (2019) of the study. In the second year, one horse became and remained WNV IgG negative for the remainder of the study period. The remaining horses were seropositive for all 5 years. The ELISA optical densities (ODs) and inhibitory percentages (IPs) of each year are presented in Table S1.

### 3.3 Virus Neutralization Assays

**FIGURE 2:**
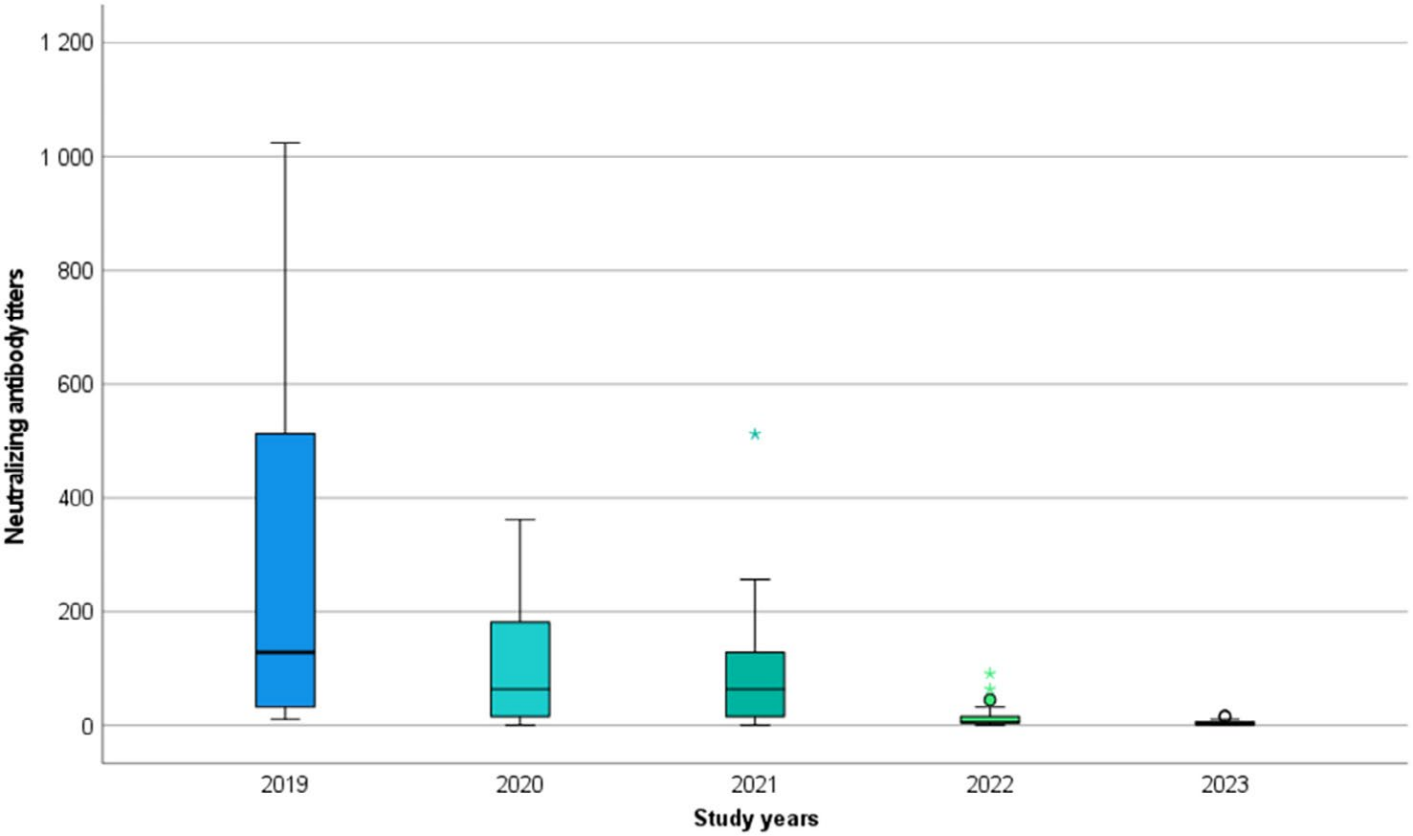
The WNV neutralizing antibody titers (*y*‐axis) of the 25 included horses during the study period (*x*‐axis). The mean WNV titer of horses was 260.64 (SD = 336.739) in 2019, 114.32 (SD = 107.36) in 2020, 95.38 (SD = 115.56) in 2021, 22.53 (SD = 25.71) in 2022 and 6.31 (SD = 5.15) in 2023. There was a significant decrease (*p* < 0.001) in the serum neutralizing antibody levels over time.

The virus‐specific neutralization antibody titers are presented in Table S2 and Figure 2.

#### 3.3.1 WNV Titers

The mean WNV titer of horses was 260.64 ± 336.739 in 2019, 114.32 ± 107.36 in 2020, 95.38 ± 115.56 in 2021, 22.53 ± 25.71 in 2022, and 6.31 ± 5.15 in 2023. A significant decrease (*p* < 0.001) in the serum VNTs occurred over time. In the first year (2019), all horses had positive WNV VNT titers; in the second year, 1/25 (4%); in the third year, 3/25 (12%); and in the fourth year, 17/25 horses (68%) had titers below the cut‐off, which increased to 22/25 (88%) in the fifth year (2023), respectively.

#### 3.3.2 USUV Titers

The mean USUV titer was 16.56 ± 34.04 in 2019, 4.16 ± 3.31 in 2020, 4.24 ± 4.68 in 2021, 4.61 ± 4.1 in 2022 and 8.71 ± 19.51 in 2023. In the first year 5/25 (20%), in the second year 1/25 (4%), in the third year 3/25 (12%), in the fourth year 2/25 (8%) and in the last year 4/25 (16%) horses had positive USUV VNT results. The USUV titers in the first 4 years of the study were not significantly different from the WNV titers, but in the last year, Horses No. 7 (1:45), No. 14 (1:64), and No. 21 (1:64) had increases in USUV VNT titer compared with the negative titers (≤ 1:8) of the previous year.

#### 3.3.3 TBEV Titers

Only 1 horse (Horse No. 6) had a positive TBEV titer of 1:128 in the first year of the study. This horse did not have detectable TBEV‐specific neutralizing antibodies in the subsequent years. The remaining horses were TBEV‐negative during the entire study period.

### 3.4 Correlation of ELISA and VNT Results

No correlations were found between the ELISA OD and IP results and the WNV VNT titers.

## 4 Discussion

In our prospective cohort study, the orthoflavivirus‐specific neutralizing antibody titers of 25 WNV naturally subclinically infected horses were followed annually from 2019 to 2023 in Hungary. Orthoflavivirus infections induce long‐term immunity in humans and birds [23, 24, 29]. According to our results, WNV infection might not provide long‐term humoral immune protection against re‐infection in naturally subclinically infected horses. We also report the first USUV seroconversion in horses in Hungary. Although VNTs are considered to be the most specific serodiagnostic methods, cross‐reactions between WNV and USUV, and in one instance, between WNV and TBEV, still were observed. Therefore, our findings emphasize the necessity of evaluating the interpretation of serological diagnostic methods in regions where multiple orthoflaviviruses are co‐circulating.

Changing climatic conditions provide favorable circumstances for the vectors of orthoflaviviruses, and urbanization, global transport, and travel further facilitate transmission of these agents [8]. The co‐circulation of orthoflaviviruses has become an emerging problem in recent years, and the number of affected areas is increasing [30]. All orthoflaviviruses show variable degrees of antigenic relatedness because of similarities in E protein amino acid sequences, allowing their classification into eight distinct serocomplexes [17]. Presently, there are 3 endemic orthoflaviviruses in central Europe: tick‐borne encephalitis virus, Usutu virus, and WNV [6, 7]. Tick‐borne encephalitis virus is a member of the mammalian tick‐borne virus complex. It was first described in 1952 in Hungary, and virus‐induced neurological disease is being reported annually among human patients, whereas an equine TBE case or seropositivity has never been reported [6]. Austria, Germany, and Switzerland have already registered TBEV‐induced neurological disease, and other European countries reported TBEV seropositivity among horses [31, 32, 33]. The Japanese encephalitis virus complex involves USUV and WNV [17]. The mosquito‐transmitted USUV was first reported in 2005 in Hungary, whereas the first USUV‐induced neurologic disease in humans was diagnosed in 2018 [34]. Usutu virus infection has never been described as causing clinical signs in horses, but seropositivity has already been reported in several European countries, but not in Hungary [35, 36, 37]. West Nile virus was first isolated in 2003 in Hungary, whereas the first West Nile neuroinvasive disorder (WNND) in horses was diagnosed in 2007 [38, 39]. Afterwards, a few cases were recorded annually; in 2016, there was a mild increase in the reported equine WNND cases. In 2018, a major WNV outbreak occurred in Hungary, as well as in many other European countries. In 2023 and 2024, the incidence of registered WNND cases in both humans and horses increased significantly, compared to previous years (European Centre for Disease Control, ECDC, 2024) [40, 41].

One of the major limitations of our study is the lack of WNV IgM‐positive ELISA results of the horses involved in this study, and thus a precise season of infection cannot be established. However, considering the substantial WNV outbreaks in Hungary (2008, 2016, 2018), and the mean age of our study population (11.5 years), we can assume that the animals were either infected in 2016 or 2018. Although the micro‐well virus neutralization test (MW‐NT) was preferred over the plaque reduction neutralization (PRNT) test because MW‐NT is more rapid and less labor intensive, and it was proposed to be an effective and reliable alternative to PRNT [24], sera samples from different years were evaluated separately because of the parallel testing for all three endemic orthoflaviviruses and the relatively high number of samples (75 VNT test samples per year). We believe this method did not importantly influence the results, because the assays were performed by the same individual using the same instruments, with both virus and cell control components included in each test. Additionally, freezing and thawing may have affected our results, but previous studies indicate that these processes do not notably affect the outcomes of VNTs and ELISAs [42, 43, 44].

After the clearance of the viral agent, long‐lived plasma cells secrete pathogen‐specific antibodies in the absence of antigen or secondary challenge for many years, whereas virus‐specific memory B‐cells (MBC) circulate in the body without antibody production. In case of a subsequent viral infection, MBCs are rapidly activated to secrete pathogen‐specific antibodies [9, 45, 46]. In orthoflaviviral infections, the concentration of neutralizing antibodies is the principal contributing factor of long‐term antiviral protection, as direct correlations were observed between in vivo neutralizing activity and in vitro protective capacity of antibodies in mice and hamster models [18, 19, 47, 48]. The neutralizing activity of antibodies is influenced by their affinity and concentration, and accessibility of viral epitopes. Strongly neutralizing antibodies have high affinity. Therefore, they can neutralize the virus in lower quantities, whereas weakly neutralizing antibodies might be unable to neutralize the agent, even in high concentrations [49]. The WNV titer of the naturally subclinically infected horses in our study decreased significantly over time (Figure 2). The primary advantage of the two‐fold serial dilution used in the VNTs of our study is that it provided more detailed monitoring of the nAb titers in comparison to the serial dilution recommended by the WOAH manual. The 1:8 titer, being closest to the WOAH positive titer of 1:10, was chosen as the cut‐off value for our study. Based on this criterion, it was found that 88% of the animals had negative WNV VNT results in 2023. Our findings are in agreement with a previous study that identified a progressive decrease in WNV‐specific IgG antibody concentrations 12–18 months post‐infection among subclinically infected human blood donors. At the same time, some patients became seronegative in a relatively short time [22]. The age of the horses in our study population is an important factor to consider regarding the decreasing antibody titers. Immunosenescence, the age‐related decline in immune function, is a well‐documented phenomenon of older horses [50, 51]. Studies have shown that geriatric horses often exhibit decreased antibody production in response to antigenic stimulation [51, 52]. Although we were unable to establish any direct correlations between the age of the horses and their antibody titers, it is essential to recognize that the life expectancy of horses has gradually increased over the past few decades [53, 54]. Therefore, further investigation into the immune responses of geriatric horses is warranted, particularly concerning their natural protection against WNV. The concentration of neutralizing antibodies that provides adequate protection against WNV re‐infection is presently unknown. However, we assume that the antibody concentrations in the last year of our study (2023) alone cannot provide protection against re‐infection [19, 55]. Furthermore, it is worth mentioning that one of the participating horses (Horse No. 5) with a WNV VNT titer of 4 in 2023 presented with mild neurological signs during the WNV transmission season in 2024. A wide range of differential diagnoses can be listed as a potential cause of mild seasonal neurological signs in horses in Europe (including TBE, equine herpes myeloencephalitis, bacterial meningoencephalitis, anaplasmosis, and neuroborreliosis) [15, 56, 57], but because this horse was kept in the north‐western part of the country (Győr‐Moson‐Sopron County) reporting one of the highest levels of WNV activity in 2024, a secondary WNV infection is also possible [42]. Although the horse tested negative for WNV IgM, animals that have already been exposed to WNV infection (or basic immunization without booster vaccines in the following years) might not develop sufficient WNV‐specific IgM during a secondary immune response to be diagnosed. The lack of or lower magnitude of virus‐specific IgM response during secondary infections is a well‐known phenomenon in dengue viral infections, pathogen of the *Orthoflavivirus* genus, important in humans [58, 59]. Concerning the antigenic relatedness of these agents, a previous orthoflavivirus infection could have hampered the diagnosis of secondary heterologous orthoflaviviral infection [17]. This possibility is supported by a study, in which horses that had previously been vaccinated against Japanese encephalitis virus (JEV) were subsequently challenged with WNV [60]. Only one horse developed a detectable WNV‐specific IgM response. Similar findings were reported in humans, where a previous WNV exposure resulted in an undetectable IgM response during secondary clinical TBEV infection [61]. Another interesting fact to consider is the lack of TBE cases in horses despite the relatively high incidence of TBE in humans in Hungary. On the other hand, seasonal neurological cases are noted among horses, as in the case of Horse No. 5, in each WNV season that test negative on WNV IgM ELISA. However, approximately half of these samples test positive on WNV IgG ELISA (Tolnai et al. unpublished data). We propose that the IgM‐based diagnostic approaches of WNV infection in countries with other co‐circulating orthoflaviviruses might be misleading. The VNT currently is recognized as the gold standard method for identifying the infecting orthoflaviviral agent. It also has the ability to overcome the limitations of the IgM ELISAs. This assay has the highest specificity among the available serodiagnostic tests because it detects the neutralizing capacity of antibodies that is influenced by their affinity, concentration, and the accessibility of the viral epitopes [49]. However, because the immunodominant protein of orthoflaviviruses is the E‐protein that possesses different levels of amino acid sequence homologies, and because WNV infections elicit a polyclonal antibody response in horses as well, cross‐reactions might occur on VNTs, especially between closely related agents [17, 62]. To minimize the risk of misleading results, it is recommended to conduct parallel VNTs for co‐circulating orthoflaviviruses. If positive results are obtained for two or more agents, the end‐point neutralization titer can help clarify any conflicting results [63, 64]. In our study population, five horses had positive USUV VNT results in 2019. Considering the significantly higher WNV titers in these animals and the genetic relatedness of these viruses, cross‐reactivity is more plausible than USUV seropositivity. In the following years, the USUV titers were insignificant in comparison to the WNV titers. In 2023, however, three horses developed a significant USUV titer increase in comparison to the previous years, as well as the WNV titer of the same year. Usutu virus is endemic in Hungary; it is regularly isolated from birds as part of a passive surveillance program [34, 65]. Neighboring countries have already reported USUV seropositivity in horses, and here, we also present evidence of subclinical USUV seroconversion in horses in Hungary [37]. The possibility of simultaneous WNV and USUV infections must be considered when interpreting the VNT results of Horses No. 1 and No. 6 in 2019, because both viruses share the same transmission cycles and vectors. However, in the case of co‐infection, we would not expect such decreases in the USUV titers in the following years. On the other hand, the persistence of USUV antibodies in horses is presently unknown. Regarding the TBEV titers, only one horse (Horse No. 6) had a positive titer in 2019. However, this horse also had positive USUV titers in 2019, and the WNV titers were significantly higher than the TBEV and USUV titers. Furthermore, the TBEV titer was negative for the remainder of the study period, whereas TBEV‐specific antibodies were reported to persist for at least 12–19 months in horses [66]. Therefore, cross‐reactivity seems to be a more relevant alternative than a simultaneous infection. Although the VNT is the most specific method for the diagnosis of orthoflaviviral infections, cross‐reactive antibodies between closely related agents such as WNV and JEV have already been reported in horses [67]. Our results support these findings and further indicate that cross‐reactivity with agents in other serocomplexes also may occur. The lack of TBEV seroconversions might be explained by the location of the stables (Figure 1) where the horses in our study were kept, because the incidence of TBE in humans is the highest in the western and southeastern parts of the country, where the humid weather conditions favor the vectors [6]. Still, the majority of the animals in our study population were kept outside of these geographic regions. Based on our results and the data in the literature [15, 31, 66], we propose that TBE in horses remains misdiagnosed in Hungary and possibly in other endemic countries because of cross‐reacting antibodies from a previous orthoflaviviral infection and the lack of direct diagnostic evaluation [61]. Alternatively, TBEV and USUV‐positive horses previously may have been exposed to these viruses, because it was shown in studies of humans that the MBC pool generated during the primary orthoflavivirus infection could recognize the related viral antigens. Consequently, upon secondary orthoflavivirus encounter, cross‐reactive antibodies to the primary agent are rapidly elicited, while the secondary virus‐specific antibody titer increases gradually. As the immune response evolves, the concentration and avidity of the antibodies against the secondary infecting agent eventually exceed that of the primary agent [9]. The data regarding the MBC response of horses in orthoflaviviral infections are scarce, but in the aforementioned study [60], the JEV‐vaccinated horses also had higher JEV titers in comparison to the WNV titers in the first 3 weeks after experimental WNV infection. In our study, the horses with positive TBEV and USUV titers in 2019 had higher WNV titers in this and subsequent years. Therefore, we believe that it is a cross‐reaction rather than a previous exposure.

A previous orthoflaviviral infection not only impedes the diagnosis but also may influence the clinical manifestations of the secondary infection. The antibody‐dependent enhancement of infections (ADE) is a well‐described phenomenon in dengue viral (DENV) infections when a person with a previous DENV infection is infected with a heterologous DENV serotype [68]. Antibody‐dependent enhancement of infection occurs when antibodies attach to the virus, because these are either present in a suboptimal concentration or are non‐neutralizing, the non‐neutralized virus is internalized by the phagocytes recruited to clear the virus‐antibody complex, which leads to enhanced viral replication within these cells [16, 69]. Although both lineage I and lineage II West Nile strains are co‐circulating in Europe, ADE has never been reported in clinically infected human or equine patients [70]. On the other hand, ADE was described in one study, where TBEV antibodies from a previous infection were shown to enhance the severity of WNV infection in mice [61, 71]. In contrast, mouse models did not show ADE in WNV infections [72, 73]. Furthermore, a primary USUV infection was protective against lethal WNV infection in geese and mice [74, 75]. The influence of a previous orthoflavivirus infection on the clinical manifestation of WNV infection in horses is currently unknown. The horses in our study did not have clinical signs after the suspected USUV infections. Although USUV has never been reported to induce clinical disease in horses, the incidence of USUV‐related neurological disease in human patients has significantly increased in recent years in Europe [37]. As the number of geographic regions where two or more orthoflaviviruses are co‐circulating increases, the influence of previous orthoflaviviral infection on the clinical manifestation of the secondary infection should be further investigated.

No correlations were found between the VNT titers and ELISA ODs or between the VNT titers and ELISA IPs. The same results were obtained from a recent study in humans and an earlier study in horses; but different ELISA kits were used [76, 77]. In our study, the INgezim WNV IgG ELISA kit was used which targets the antibodies produced against a specific epitope of the E protein of WNV. The antibodies in the serum sample compete against the recombinant monoclonal antibody (mAb) for the epitope‐binding site. As already mentioned, the horse's anti‐WNV antibody response is polyclonal, and therefore antibodies are produced against many epitopes on the E protein [62]. As mentioned above, the neutralization activity depends on the antibodies' affinity and concentration. Non‐neutralizing or low‐affinity antibodies can be attached to the epitope, which is recognized by the recombinant mAb used in the kit [78]. Hence, considering the specificity of the E‐protein IgG ELISAs and the lack of correlation between the neutralizing titers and IPs, the relevance of IgG ELISA results without VNT confirmation in the evaluation of protection is not recommended.

Inactivated and recombinant canarypox‐vector vaccines are available for horses for use against WNV. To date, only one study has evaluated the antibody response of WNV seropositive horses in response to immunization with one of the inactivated vaccines [79]. Seropositive horses mounted significant antibody titer increases even after the first vaccine of the base immunization, in comparison to the seronegative animals [80]. Based on the antibody titers of the horses in the fifth year of our study, we recommend consideration of immunization of naturally WNV subclinically infected horses.

In summary, WNV seroconvalescence in horses elicits a virus‐specific neutralizing antibody response, but the WNV titers decrease significantly over time. Previous exposure and co‐circulation of orthoflaviviruses can substantially influence the serodiagnosis of orthoflaviviral infections. Based on our findings, we advise VNTs conducted in parallel for co‐circulating agents. To achieve optimal humoral immunoprotection, we recommend regular monitoring of antibody titers of WNV seroconverted horses in endemic areas and vaccination of animals with negligible antibody titers.

## Disclosure

Authors declare no off‐label use of antimicrobials.

## Ethics Statement

Authors declare no institutional animal care and use committee or other approval was needed. Authors declare human ethics approval was not needed.

## Conflicts of Interest

The authors declare no conflicts of interest.

## Supporting information


Table S1.

Table S2.

## References

[1] T. S. Postler , M. Beer , B. J. Blitvich , et al., “Renaming of the Genus Flavivirus to Orthoflavivirus and Extension of Binomial Species Names Within the Family Flaviviridae,” Archives of Virology 168, no. 9 (2023): 224. 37561168 10.1007/s00705-023-05835-1

[2] J. J. Sejvar , “West Nile Virus: An Historical Overview,” Ochsner Journal 5, no. 3 (2003): 6–10. PMC3111838 21765761

[3] L. R. Petersen and E. B. Hayes , “West Nile Virus in the Americas,” Medical Clinics of North America 92, no. 6 (2008): 1307–1322. 19145778 10.1016/j.mcna.2008.07.004

[4] V. Sambri , M. Capobianchi , R. Charrel , et al., “West Nile Virus in Europe: Emergence, Epidemiology, Diagnosis, Treatment, and Prevention,” Clinical Microbiology and Infection 19, no. 8 (2013): 699–704. 23594175 10.1111/1469-0691.12211

[5] “Genus: Orthoflavivirus|ICTV [Internet],” https://ictv.global/report/chapter/flaviviridae/flaviviridae/orthoflavivirus.

[6] L. Egyed , A. Nagy , A. Lakos , V. Zöldi , and Z. Lang , “Tick‐Borne Encephalitis Epidemic in Hungary 1951‐2021: The Story and Lessons Learned,” Zoonoses and Public Health 70, no. 1 (2023): 81–92. 36205381 10.1111/zph.13003

[7] Y. Simonin , “Circulation of West Nile Virus and Usutu Virus in Europe: Overview and Challenges,” Viruses 16, no. 4 (2024): 599. 38675940 10.3390/v16040599 PMC11055060

[8] C. Chancey , A. Grinev , E. Volkova , and M. Rios , “The Global Ecology and Epidemiology of West Nile Virus,” BioMed Research International 2015, no. 1 (2015): 376230. 25866777 10.1155/2015/376230 PMC4383390

[9] A. O. Uhuami , N. Lawal , M. B. Bello , and M. U. Imam , “Flavivirus Cross‐Reactivity: Insights Into e‐Protein Conservancy, Pre‐Existing Immunity, and Co‐Infection,” Microbe 4 (2024): 100105.

[10] E. R. Schwarz and M. T. Long , “Comparison of West Nile Virus Disease in Humans and Horses: Exploiting Similarities for Enhancing Syndromic Surveillance,” Viruses 15, no. 6 (2023): 1230. 37376530 10.3390/v15061230 PMC10303507

[11] O. E. Fehér , P. Fehérvári , C. H. Tolnai , et al., “Epidemiology and Clinical Manifestation of West Nile Virus Infections of Equines in Hungary, 2007–2020,” Viruses 14, no. 11 (2022): 2551. 36423160 10.3390/v14112551 PMC9694158

[12] I. García‐Bocanegra , J. Belkhiria , S. Napp , D. Cano‐Terriza , S. Jiménez‐Ruiz , and B. Martínez‐López , “Epidemiology and Spatio‐Temporal Analysis of West Nile Virus in Horses in Spain Between 2010 and 2016,” Transboundary and Emerging Diseases 65, no. 2 (2018): 567–577. 29034611 10.1111/tbed.12742

[13] C. Faverjon , M. G. Andersson , A. Decors , et al., “Evaluation of a Multivariate Syndromic Surveillance System for West Nile Virus,” Vector Borne and Zoonotic Diseases 16, no. 6 (2016): 382–390. 27159212 10.1089/vbz.2015.1883 PMC4884334

[14] M. B. Porter , M. T. Long , L. M. Getman , et al., “West Nile Virus Encephalomyelitis in Horses: 46 Cases (2001),” 2003, https://avmajournals.avma.org/view/journals/javma/222/9/javma.2003.222.1241.xml. 10.2460/javma.2003.222.1241 12725313

[15] J. M. V. Cavalleri , O. Korbacska‐Kutasi , A. Leblond , et al., “European College of Equine Internal Medicine Consensus Statement on Equine Flaviviridae Infections in Europe,” Journal of Veterinary Internal Medicine 36, no. 6 (2022): 1858–1871. 36367340 10.1111/jvim.16581 PMC9708432

[16] K. R. Chan , A. A. Ismail , G. Thergarajan , et al., “Serological Cross‐Reactivity Among Common Flaviviruses,” Frontiers in Cellular and Infection Microbiology 12 (2022): e‑975398, https://www.frontiersin.org/journals/cellular‐and‐infection‐microbiology/articles/10.3389/fcimb.2022.975398/full. 10.3389/fcimb.2022.975398 PMC9519894 36189346

[17] A. P. S. Rathore and A. L. St. John , “Cross‐Reactive Immunity Among Flaviviruses,” Frontiers in Immunology 11, no. 334 (2020): e‑334. 10.3389/fimmu.2020.00334 PMC7054434 32174923

[18] J. T. Roehrig , L. A. Staudinger , A. R. Hunt , J. H. Mathews , and C. D. Blair , “Antibody Prophylaxis and Therapy for Flavivirus Encephalitis Infections,” Annals of the New York Academy of Sciences 951 (2001): 286–297. 11797785 10.1111/j.1749-6632.2001.tb02704.x

[19] R. B. Tesh , A. P. A. Travassos Da Rosa , H. Guzman , T. P. Araujo , and S. Y. Xiao , “Immunization With Heterologous Flaviviruses Protective Against Fatal West Nile Encephalitis,” Emerging Infectious Diseases 8, no. 3 (2002): 245–251. 11927020 10.3201/eid0803.010238 PMC2732478

[20] T. R. Kreil , E. Maier , S. Fraiss , and M. M. Eibl , “Neutralizing Antibodies Protect Against Lethal Flavivirus Challenge but Allow for the Development of Active Humoral Immunity to a Nonstructural Virus Protein,” Journal of Virology 72, no. 4 (1998): 3076–3081. 9525632 10.1128/jvi.72.4.3076-3081.1998 PMC109757

[21] P. Kaaijk and W. Luytjes , “Are We Prepared for Emerging Flaviviruses in Europe? Challenges for Vaccination,” Human Vaccines & Immunotherapeutics 14, no. 2 (2017): 337–344. 29053401 10.1080/21645515.2017.1389363 PMC5806644

[22] A. Pierro , P. Gaibani , C. Spadafora , et al., “Detection of Specific Antibodies Against West Nile and Usutu Viruses in Healthy Blood Donors in Northern Italy, 2010–2011,” Clinical Microbiology and Infection 19, no. 10 (2013): E451–E453. 23663225 10.1111/1469-0691.12241

[23] P. J. Carson , H. E. Prince , B. J. Biggerstaff , R. Lanciotti , L. H. Tobler , and M. Busch , “Characteristics of Antibody Responses in West Nile Virus‐Seropositive Blood Donors,” Journal of Clinical Microbiology 52, no. 1 (2020): 57–60. 10.1128/JCM.01932-13 PMC3911469 24131687

[24] A. Papa , A. Anastasiadou , and M. Delianidou , “West Nile Virus IgM and IgG Antibodies Three Years Post‐ Infection,” Hippokratia 19, no. 1 (2015): 34–36. 26435644 PMC4574583

[25] “West Nile Virus Strain 578/10, Complete Genome,” (2014), http://www.ncbi.nlm.nih.gov/nuccore/KC496015.1.

[26] “Usutu Virus Isolate Budapest, Complete Genome—Nucleotide—NCBI [Internet],” https://www.ncbi.nlm.nih.gov/nucleotide/EF206350.1?report=genbank&log$=nucltop&blast_rank=18&RID=MDB6SRWT013.

[27] “Tick‐Borne Encephalitis Virus Isolate KEM‐1, Complete Genome [Internet],” 2021, http://www.ncbi.nlm.nih.gov/nuccore/MW256716.1.

[28] “3. 01.25_WEST_NILE.pdf [Internet],” https://www.woah.org/fileadmin/Home/fr/Health_standards/tahm/3.01.25_WEST_NILE.pdf.

[29] N. M. Nemeth , P. T. Oesterle , and R. A. Bowen , “Humoral Immunity to West Nile Virus Is Long‐Lasting and Protective in the House Sparrow ( *Passer domesticus* ),” American Journal of Tropical Medicine and Hygiene 80, no. 5 (2009): 864–869. 19407139 PMC2693945

[30] T. C. Pierson and M. S. Diamond , “The Continued Threat of Emerging Flaviviruses,” Nature Microbiology 5, no. 6 (2020): 796–812. 10.1038/s41564-020-0714-0 PMC7696730 32367055

[31] P. de Heus , Z. Bagó , P. Weidinger , et al., “Severe Neurologic Disease in a Horse Caused by Tick‐Borne Encephalitis Virus, Austria, 2021,” Viruses 15, no. 10 (2023): 2022. 37896799 10.3390/v15102022 PMC10611255

[32] N. Fouché , S. Oesch , U. Ziegler , and V. Gerber , “Clinical Presentation and Laboratory Diagnostic Work‐Up of a Horse With Tick‐Borne Encephalitis in Switzerland,” Viruses 13, no. 8 (2021): 1474. 34452340 10.3390/v13081474 PMC8402657

[33] D. Kälin , A. Becsek , H. Stürmer , et al., “Immune Response After Vaccination Against Tick‐Borne Encephalitis Virus (TBEV) in Horses,” Vaccine 12, no. 9 (2024): 1074. 10.3390/vaccines12091074 PMC11435670 39340104

[34] A. Nagy , E. Mezei , O. Nagy , et al., “Extraordinary Increase in West Nile Virus Cases and First Confirmed Human Usutu Virus Infection in Hungary, 2018,” Eurosurveillance 24, no. 28 (2019): 1900038. 31311619 10.2807/1560-7917.ES.2019.24.28.1900038 PMC6636212

[35] T. Vilibic‐Cavlek , T. Petrovic , V. Savic , et al., “Epidemiology of Usutu Virus: The European Scenario,” Pathogens 9, no. 9 (2020): 699. 32858963 10.3390/pathogens9090699 PMC7560012

[36] D. Cadar and Y. Simonin , “Human Usutu Virus Infections in Europe: A New Risk on Horizon?,” Viruses 15, no. 1 (2023): 77. 10.3390/v15010077 PMC9866956 36680117

[37] G. Angeloni , M. Bertola , E. Lazzaro , et al., “Epidemiology, Surveillance and Diagnosis of Usutu Virus Infection in the EU/EEA, 2012 to 2021,” Eurosurveillance 28, no. 33 (2023): 2200929. 37589592 10.2807/1560-7917.ES.2023.28.33.2200929 PMC10436690

[38] O. Kutasi , T. Bakonyi , S. Lecollinet , et al., “Equine Encephalomyelitis Outbreak Caused by a Genetic Lineage 2 West Nile Virus in Hungary,” Veterinary Internal Medicne 25, no. 3 (2011): 586–591. 10.1111/j.1939-1676.2011.0715.x 21457323

[39] T. Bakonyi , É. Ivanics , K. Erdélyi , et al., “Lineage 1 and 2 Strains of Encephalitic West Nile Virus, Central Europe,” Emerging Infectious Diseases 12, no. 4 (2006): 618–623. 16704810 10.3201/eid1204.051379 PMC3294705

[40] “Epidemiological Update: West Nile Virus Transmission Season in Europe, 2023 [Internet],” (2024), https://www.ecdc.europa.eu/en/news‐events/epidemiological‐update‐west‐nile‐virus‐transmission‐season‐europe‐2023‐0.

[41] “Monthly Updates: 2024 West Nile Virus Transmission Season [Internet],” (2024), https://www.ecdc.europa.eu/en/infectious‐disease‐topics/west‐nile‐virus‐infection/surveillance‐and‐disease‐data/monthly‐updates.

[42] G. Kuno , “Serodiagnosis of Flaviviral Infections and Vaccinations in Humans,” in Advances in Virus Research (Elsevier, 2003), 3–65, https://linkinghub.elsevier.com/retrieve/pii/S0065352703610018. 10.1016/s0065-3527(03)61001-8 14714429

[43] A. Torelli , E. Gianchecchi , M. Monti , et al., “Effect of Repeated Freeze–Thaw Cycles on Influenza Virus Antibodies,” Vaccine 9, no. 3 (2021): 267. 10.3390/vaccines9030267 PMC8002830 33802846

[44] F. M. Shurrab , D. W. Al‐Sadeq , F. Amanullah , et al., “Effect of Multiple Freeze–Thaw Cycles on the Detection of Anti‐SARS‐CoV‐2 IgG Antibodies,” Journal of Medical Microbiology 70, no. 8 (2021): 001402. 34356000 10.1099/jmm.0.001402 PMC8513627

[45] R. Wong , J. A. Belk , J. Govero , et al., “Affinity‐Restricted Memory B Cells Dominate Recall Responses to Heterologous Flaviviruses,” Immunity 53, no. 5 (2020): 1078–1094.e7. 33010224 10.1016/j.immuni.2020.09.001 PMC7677180

[46] A. Adam , S. Cuellar , and T. Wang , “Memory B Cell and Antibody Responses to Flavivirus Infection and Vaccination,” Faculty Reviews 10 (2021): 5. 33659923 10.12703/r/10-5 PMC7894259

[47] T. Oliphant , G. E. Nybakken , M. Engle , et al., “Antibody Recognition and Neutralization Determinants on Domains I and II of West Nile Virus Envelope Protein,” Journal of Virology 80, no. 24 (2006): 12149–12159. 17035317 10.1128/JVI.01732-06 PMC1676294

[48] T. C. Pierson , Q. Xu , S. Nelson , et al., “The Stoichiometry of Antibody‐Mediated Neutralization and Enhancement of West Nile Virus Infection,” Cell Host & Microbe 1, no. 2 (2007): 135–145. 18005691 10.1016/j.chom.2007.03.002 PMC2656919

[49] M. S. Diamond , T. C. Pierson , and D. H. Fremont , “The Structural Immunology of Antibody Protection Against West Nile Virus,” Immunological Reviews 225, no. 1 (2008): 212–225. 18837784 10.1111/j.1600-065X.2008.00676.x PMC2646609

[50] D. W. Horohov , A. A. Adams , and T. M. Chambers , “Immunosenescence of the Equine Immune System,” Journal of Comparative Pathology 142 (2010): S78–S84. 19897209 10.1016/j.jcpa.2009.10.007

[51] S. DeNotta and D. McFarlane , “Immunosenescence and Inflammaging in the Aged Horse,” Immunity & Ageing 20, no. 1 (2023): 2. 36609345 10.1186/s12979-022-00325-5 PMC9817422

[52] S. Hansen , K. E. Baptiste , J. Fjeldborg , and D. W. Horohov , “A Review of the Equine Age‐Related Changes in the Immune System: Comparisons Between Human and Equine Aging, With Focus on Lung‐Specific Immune‐Aging,” Ageing Research Reviews 20 (2015): 11–23. 25497559 10.1016/j.arr.2014.12.002

[53] C. E. Welsh , M. Duz , T. D. H. Parkin , and J. F. Marshall , “Prevalence, Survival Analysis and Multimorbidity of Chronic Diseases in the General Veterinarian‐Attended Horse Population of the UK,” Preventive Veterinary Medicine 131, no. 1 (2016): 137–145. 27544263 10.1016/j.prevetmed.2016.07.011

[54] E. M. Rankins , C. L. Wickens , K. H. McKeever , and K. Malinowski , “A Survey of Horse Selection, Longevity, and Retirement in Equine‐Assisted Services in the United States,” Animals 11, no. 8 (2021): 2333. 34438791 10.3390/ani11082333 PMC8388649

[55] A. H. Davidson , J. L. Traub‐Dargatz , R. M. Rodeheaver , et al., “Immunologic Responses to West Nile Virus in Vaccinated and Clinically Affected Horses,” Journal of the American Veterinary Medical Association 226, no. 2 (2005): 240–245. 15706975 10.2460/javma.2005.226.240

[56] S. Lecollinet , S. Pronost , M. Coulpier , et al., “Viral Equine Encephalitis, a Growing Threat to the Horse Population in Europe?,” Viruses 12, no. 1 (2019): 23. 31878129 10.3390/v12010023 PMC7019608

[57] N. Kašpárková , E. Bártová , A. Žákovská , M. Budíková , and K. Sedlák , “Antibodies Against *Borrelia burgdorferi* Sensu Lato in Clinically Healthy and Sick Horses: First Report From The Czech Republic,” Microorganisms 11, no. 7 (2023): 1706. 37512879 10.3390/microorganisms11071706 PMC10386530

[58] A. L. St. John and A. P. S. Rathore , “Adaptive Immune Responses to Primary and Secondary Dengue Virus Infections,” Nature Reviews. Immunology 19, no. 4 (2019): 218–230. 10.1038/s41577-019-0123-x 30679808

[59] S. Chanama , S. Anantapreecha , A. A‐nuegoonpipat , A. Sa‐gnasang , I. Kurane , and P. Sawanpanyalert , “Analysis of Specific IgM Responses in Secondary Dengue Virus Infections: Levels and Positive Rates in Comparison With Primary Infections,” Journal of Clinical Virology 31, no. 3 (2004): 185–189. 15465410 10.1016/j.jcv.2004.03.005

[60] H. Shirafuji , K. Kanehira , T. Kamio , et al., “Antibody Responses Induced by Experimental West Nile Virus Infection With or Without Previous Immunization With Inactivated Japanese Encephalitis Vaccine in Horses,” Journal of Veterinary Medical Science 71, no. 7 (2009): 969–974. 19652487 10.1292/jvms.71.969

[61] E. Ferenczi , E. Bán , A. Ábrahám , et al., “Severe Tick‐Borne Encephalitis in a Patient Previously Infected by West Nile Virus,” Scandinavian Journal of Infectious Diseases 40, no. 9 (2008): 759–761. 19086342 10.1080/00365540801995386

[62] M. D. Sánchez , T. C. Pierson , M. M. DeGrace , et al., “The Neutralizing Antibody Response Against West Nile Virus in Naturally Infected Horses,” Virology 359, no. 2 (2007): 336–348. 17055550 10.1016/j.virol.2006.08.047

[63] F. A. Rey , K. Stiasny , M. Vaney , M. Dellarole , and F. X. Heinz , “The Bright and the Dark Side of Human Antibody Responses to Flaviviruses: Lessons for Vaccine Design,” EMBO Reports 19, no. 2 (2018): 206–224. 29282215 10.15252/embr.201745302 PMC5797954

[64] F. Llorente , A. García‐Irazábal , E. Pérez‐Ramírez , et al., “Influence of Flavivirus Co‐Circulation in Serological Diagnostics and Surveillance: A Model of Study Using West Nile, Usutu and Bagaza Viruses,” Transboundary and Emerging Diseases 66, no. 5 (2019): 2100–2106. 31150146 10.1111/tbed.13262

[65] A. Nagy , N. Csonka , M. Takács , E. Mezei , and É. Barabás , “West Nile and Usutu Virus Seroprevalence in Hungary: A Nationwide Serosurvey Among Blood Donors in 2019,” PLoS One 17, no. 4 (2022): e0266840. 35395048 10.1371/journal.pone.0266840 PMC8992992

[66] C. Klaus , U. Hörügel , B. Hoffmann , and M. Beer , “Tick‐Borne Encephalitis Virus (TBEV) Infection in Horses: Clinical and Laboratory Findings and Epidemiological Investigations,” Veterinary Microbiology 163, no. 3 (2013): 368–372. 23395291 10.1016/j.vetmic.2012.12.041

[67] J. Hirota , H. Nishi , H. Matsuda , H. Tsunemitsu , and S. Shimiz , “Cross‐Reactivity of Japanese Encephalitis Virus‐Vaccinated Horse Sera in Serodiagnosis of West Nile Virus,” Journal of Veterinary Medical Science 72, no. 3 (2010): 369–372. 19996564 10.1292/jvms.09-0311

[68] J. Flipse , M. A. Diosa‐Toro , T. E. Hoornweg , D. P. I. van de Pol , S. Urcuqui‐Inchima , and J. M. Smit , “Antibody‐Dependent Enhancement of Dengue Virus Infection in Primary Human Macrophages; Balancing Higher Fusion Against Antiviral Responses,” Scientific Reports 6, no. 1 (2016): 29201. 27380892 10.1038/srep29201 PMC4933910

[69] R. Wong and D. Bhattacharya , “Basics of Memory B‐Cell Responses: Lessons From and for the Real World,” Immunology 156, no. 2 (2019): 120–129. 30488482 10.1111/imm.13019 PMC6328991

[70] L. Barzon , F. Montarsi , E. Quaranta , et al., “Early Start of Seasonal Transmission and Co‐Circulation of West Nile Virus Lineage 2 and a Newly Introduced Lineage 1 Strain, Northern Italy, June 2022,” Eurosurveillance 27, no. 29 (2022): 2200548. 35866436 10.2807/1560-7917.ES.2022.27.29.2200548 PMC9306260

[71] R. J. Phillpotts , J. R. Stephenson , and J. S. Porterfield , “Antibody‐Dependent Enhancement of Tick‐Borne Encephalitis Virus Infectivity,” Journal of General Virology 66, no. Pt 8 (1985): 1831–1837. 2991448 10.1099/0022-1317-66-8-1831

[72] M. R. Vogt , K. A. Dowd , M. Engle , et al., “Poorly Neutralizing Cross‐Reactive Antibodies Against the Fusion Loop of West Nile Virus Envelope Protein Protect In Vivo via Fcγ Receptor and Complement‐Dependent Effector Mechanisms,” Journal of Virology 85, no. 22 (2011): 11567–11580. 21917960 10.1128/JVI.05859-11 PMC3209272

[73] H. Sun , D. Acharya , A. M. Paul , et al., “Antibody‐Dependent Enhancement Activity of a Plant‐Made Vaccine Against West Nile Virus,” Vaccine 11, no. 2 (2023): 197. 10.3390/vaccines11020197 PMC9966755 36851075

[74] H. Reemtsma , C. M. Holicki , C. Fast , F. Bergmann , M. H. Groschup , and U. Ziegler , “A Prior Usutu Virus Infection Can Protect Geese From Severe West Nile Disease,” Pathogens 12, no. 7 (2023): 959. 37513806 10.3390/pathogens12070959 PMC10386565

[75] A. B. Blázquez , E. Escribano‐Romero , M. A. Martín‐Acebes , T. Petrovic , and J. C. Saiz , “Limited Susceptibility of Mice to Usutu Virus (USUV) Infection and Induction of Flavivirus Cross‐Protective Immunity,” Virology 482 (2015): 67–71. 25827530 10.1016/j.virol.2015.03.020

[76] T. Vilibic‐Cavlek , M. Bogdanic , V. Savic , et al., “Diagnosis of West Nile Virus Infections: Evaluation of Different Laboratory Methods,” World Journal of Virology 13, no. 4 (2024): 95986, https://www.wjgnet.com/2220‐3249/full/v13/i4/95986.htm. 39722752 10.5501/wjv.v13.i4.95986 PMC11551685

[77] T. Oliphant , G. E. Nybakken , S. K. Austin , et al., “Induction of Epitope‐Specific Neutralizing Antibodies Against West Nile Virus,” Journal of Virology 81, no. 21 (2007): 11828–11839. 17715236 10.1128/JVI.00643-07 PMC2168772

[78] E. Sotelo , F. Llorente , B. Rebollo , et al., “Development and Evaluation of a New Epitope‐Blocking ELISA for Universal Detection of Antibodies to West Nile Virus,” Journal of Virological Methods 174, no. 1–2 (2011): 35–41. 21419800 10.1016/j.jviromet.2011.03.015

[79] F. Monaco , G. Purpari , A. Di Gennaro , et al., “Immunological Response in Horses Following West Nile Virus Vaccination With Inactivated or Recombinant Vaccine,” Veterinaria Italiana 55, no. 1 (2019): 73–79. 30951184

[80] K. Joó , T. Bakonyi , O. Szenci , et al., “Comparison of Assays for the Detection of West Nile Virus Antibodies in Equine Serum After Natural Infection or Vaccination,” Veterinary Immunology and Immunopathology 183 (2017): 1–6. 28063471 10.1016/j.vetimm.2016.10.015

