# Supplementary material for: Long‐Term Humoral Immune Response After West Nile Virus Convalescence in Horses in a Geographic Area of Multiple Orthoflavivirus Co‐Circulation
Source: J Vet Intern Med. 2025 Jun 17;39(4):e70176. doi: 10.1111/jvim.70176 (PMC12171932; doi:10.1111/jvim.70176)
Supplement: Supplementary file 1 — Table S1. [file JVIM-39-e70176-s002.pdf]

WNV IgG ELISA 2019-2023										
Horse ID	**2019 OD**	**2019 IP**	**2020 OD**	**2020 IP**	**2021 OD**	**2021 IP**	**2022 OD**	**2022 IP**	**2023 OD**	**2023 IP**
**1**	0.069	95.185	0.083	94.208	0.094	93.336	0.1	93.371	0.097	91.178
**2**	0.082	94.278	0.066	95.394	0.064	95.463	0.058	96.155	0.064	94.179
**3**	0.068	95.255	0.068	95.255	0.061	95.675	0.053	96.487	0.059	94.634
**4**	0.092	93.58	0.086	93.999	0.087	93.832	0.064	95.757	0.076	93.088
**5**	0.086	93.999	0.081	94.348	0.106	92.485	0.054	96.42	0.062	94.361
**6**	0.076	94.696	0.07	95.115	0.067	95.25	0.059	96.089	0.065	94.088
**7**	0.072	94.976	0.077	94.627	0.076	94.612	0.069	95.426	0.093	91.542
**8**	0.078	94.557	0.089	93.789	0.074	94.754	0.062	95.89	0.086	92.178
**9**	0.072	94.976	0.082	94.278	0.06	95.746	0.057	96.221	0.152	86.176
**10**	0.072	94.976	0.078	94.557	0.074	94.754	0.062	95.89	0.077	92.997
**11**	0.084	94.138	0.09	93.719	0.148	89.507	0.071	95.293	0.052	95.271
**12**	0.082	94.278	0.08	94.417	0.103	92.698	0.053	96.487	0.065	94.088
**13**	0.099	93.091	0.094	93.44	0.106	92.485	0.055	96.354	0.154	85.994
**14**	0.193	86.532	0.228	84.089	0.209	85.183	0.193	87.206	0.065	94.088
**15**	0.072	94.976	0.098	93.161	0.08	94.328	0.058	96.155	0.076	93.088
**16**	0.085	94.068	0.099	93.091	0.097	93.123	0.07	95.36	0.086	92.178
**17**	0.072	94.976	0.074	94.836	0.069	95.108	0.053	96.487	0.06	94.543
**18**	0.071	95.045	0.076	94.696	0.249	82.347	0.056	96.288	0.072	93.452
**19**	0.093	93.51	0.165	88.486	0.113	91.989	0.128	91.515	0.096	91.269
**20**	0.075	94.766	0.089	93.789	0.07	95.037	0.068	95.492	0.064	94.179
**21**	0.074	94.836	0.085	94.068	0.199	85.892	0.061	95.956	0.068	93.815
**22**	0.083	94.208	0.092	93.58	0.068	95.179	0.055	96.354	0.056	94.907
**23**	0.117	91.835	0.133	90.719	0.164	88.373	0.168	88.863	0.128	88.358
**24**	0.082	94.278	1.185	17.306	1.232	12.655	1.421	5.8	1.305	-18.69
**25**	0.069	95.185	0.085	94.068	0.07	95.037	0.061	95.956	0.074	93.27

Supplemental Table 1. WNV IgG ELISA (enzyme-linked immunosorbent assay) results: optical density (OD) and inhibitory percentage (IP) of each serum sample. IP<30 - negative; IP 30-40 - doubtful, IP>40 – positive.
