# Supplementary material for: Long‐Term Humoral Immune Response After West Nile Virus Convalescence in Horses in a Geographic Area of Multiple Orthoflavivirus Co‐Circulation
Source: J Vet Intern Med. 2025 Jun 17;39(4):e70176. doi: 10.1111/jvim.70176 (PMC12171932; doi:10.1111/jvim.70176)
Supplement: Supplementary file 2 — Table S2. [file JVIM-39-e70176-s001.pdf]

Virus neutralization test titers															
	**2019**			**2020**			**2021**			**2022**			**2023**		
Horse ID	WNV	USUV	TBEV	WNV	USUV	TBEV	WNV	USUV	TBEV	WNV	USUV	TBEV	WNV	USUV	TBEV
**1**	1024	128	0	128	8	0	64	2	0	4	0	0	2	0	0
**2**	128	8	0	256	8	0	512	4	0	11	8	0	4	8	0
**3**	1024	4	0	362	4	0	256	8	0	5.7	8	0	2	4	0
**4**	256	4	0	256	2	0	256	2	0	23	0	0	8	0	0
**5**	64	8	0	128	4	0	128	11	0	16	8	0	4	0	0
**6**	512	128	128	128	16	0	128	16	0	91	8	0	16	0	0
**7**	16	8	0	16	4	0	16	2	0	2.8	0	0	1.4	45	0
**8**	32	8	0	16	2	0	23	2	0	4	8	0	1.4	0	0
**9**	128	8	0	32	4	0	64	16	0	45	8	0	11	0	0
**10**	32	8	0	16	2	0	8	2	0	2.8	2.8	0	1.4	0	0
**11**	16	4	0	64	2	0	91	2	0	5.7	2.8	0	4	2	0
**12**	16	2	0	11	2	0	5.7	8	0	5.7	4	0	1.4	0	0
**13**	32	4	0	32	2	0	45	1	0	8	0	0	2	0	0
**14**	32	8	0	32	8	0	23	8	0	16	8	0	5.7	64	0
**15**	256	11	0	256	2	0	128	0	0	8	2	0	4	2.8	0
**16**	11	4	0	11	2	0	16	2	0	1.4	2	0	1.4	0	0
**17**	128	4	0	181	2	0	181	0	0	8	0	0	4	0	0
**18**	512	4	0	256	4	0	128	0	0	64	11	0	16	16	0
**19**	1024	2	0	256	2	0	11	0	0	32	11	0	11	0	0
**20**	11	4	0	16	4	0	8	2	0	1.4	0	0	1.4	2	0
**21**	512	32	0	128	8	0	91	8	0	5.7	8	0	11	64	0
**22**	32	4	0	64	4	0	16	2	0	5.7	4	0	1.4	0	0
**23**	128	11	0	32	4	0	32	4	0	1.4	0	0	1.4	0	0
**24**	64	4	0	0	0	0	0	0	0	0	8	0	0	4	0
**25**	526	4	0	181	4	0	64	4	0	8	0	0	2	0	0

Supplemental Table 2. Virus neutralization test (VNT) titers of horses against WNV (West Nile virus), Usutu virus (USUV), and tick-borne encephalitis virus (TBEV) from 2019 to 2023.
